# Overexpression of MET is a new predictive marker for anti-EGFR therapy in metastatic colorectal cancer with wild-type *KRAS*

**DOI:** 10.1007/s00280-014-2401-4

**Published:** 2014-02-06

**Authors:** Tomokazu Kishiki, Hiroaki Ohnishi, Tadahiko Masaki, Kouki Ohtsuka, Yasuo Ohkura, Jyunji Furuse, Takashi Watanabe, Masanori Sugiyama

**Affiliations:** 1 Department of Surgery, Kyorin University School of Medicine, 6-20-2 Shinkawa, Mitaka, Tokyo 181-8611 Japan; 2 Department of Laboratory Medicine, Kyorin University School of Medicine, Tokyo, Japan; 3 Department of Pathology, Kyorin University School of Medicine, Tokyo, Japan; 4 Department of Medical Oncology, Kyorin University School of Medicine, Tokyo, Japan

**Keywords:** Colorectal cancer, *BRAF*, *PIK3CA*, PTEN, MET, Anti-EGFR therapy

## Abstract

**Purpose:**

Since the *KRAS* mutation is not responsible for all metastatic colorectal cancer (mCRC) patients with resistance to anti-epidermal growth factor receptor (EGFR) monoclonal antibody (MoAb) therapy, new predictive and prognostic factors are actively being sought.

**Methods:**

We retrospectively evaluated the efficacy of anti-EGFR MoAb-based therapies in 91 patients with mCRC according to *KRAS*, *BRAF*, and *PIK3CA* mutational status as well as PTEN and MET expression.

**Results:**

In the patient group with wild-type *KRAS*, the presence of *BRAF* mutation or *PIK3CA* mutations was associated with lower disease control rate (DCR), shorter progression-free survival (PFS), and shorter overall survival. Patients with MET overexpression also showed lower DCR and shorter PFS when compared with patients with normal MET expression. In a separate analysis, 44 patients harboring wild-type *KRAS* tumors were sorted into subgroups of 25 patients without abnormality in three molecules (*BRAF*, *PIK3CA* and MET) and 19 patients with abnormality in at least one of these three molecules. The former group showed significantly higher DCR and longer PFS following anti-EGFR therapy than the latter group.

**Conclusions:**

Our data point to the usefulness of MET overexpression, in addition to *BRAF* and *PIK3CA* mutations, as a new predictive marker for responsiveness to anti-EGFR MoAbs in mCRC patients with wild-type *KRAS*. This study also suggests that application of multiple biomarkers is more effective than the use of a single marker in selecting patients who might benefit from anti-EGFR therapy.

**Electronic supplementary material:**

The online version of this article (doi:10.1007/s00280-014-2401-4) contains supplementary material, which is available to authorized users.

## Introduction

Cetuximab and panitumumab are monoclonal antibodies (MoAbs) that inhibit the activation of the epidermal growth factor receptor (EGFR) and its downstream pathways, namely the RAS/RAF/MAPK and the PI3K/PTEN/Akt axes [1, 2]. As the response rate (RR) to anti-EGFR MoAbs remains as low as 10–20 % in patients with metastatic colorectal cancer (mCRC) [2], several studies have been performed to identify markers predicting the efficacy of these agents. Tumors carrying oncogenic *KRAS* mutations typically do not respond to anti-EGFR MoAbs therapy [3]. This finding led the European Medicines Agency and, subsequently, the US Food and Drug Administration to limit the use of cetuximab and panitumumab only to patients with wild-type *KRAS* tumors [4]. However, since only 40–60 % of patients with wild-type *KRAS* tumors respond to anti-EGFR MoAb therapy, new predictive and prognostic factors are actively being sought [5, 6]. In this regard, the presence of oncogenic deregulation of EGFR and other members of its downstream signaling pathways, such as *BRAF*, *PIK3CA*, and PTEN, has been shown to influence the responsiveness to cetuximab and panitumumab and could, therefore, help to identify nonresponder patients [4, 6–10]. While many studies have demonstrated the *BRAF* mutation, *PIK3CA* mutation, and PTEN overexpression as markers for resistance to anti-EGFR MoAb therapy, some failed to show such association [4, 7, 8, 10–13]. Therefore, analysis of these genetic markers in different patient populations, in particular in different ethnic groups, will help determine their clinical significance.

Furthermore, recent studies also have suggested that activation of MET, a tyrosine kinase that acts as a receptor for hepatocyte growth factor (HGF) and can activate the RAS/RAF/MAPK and PTEN/PI3K/Akt pathways, may be a novel mechanism of cetuximab resistance in CRC [13–18]. However, it remains unclear whether MET activation can serve as a predictive marker for the response to the anti-EGFR therapy in patients with wild-type *KRAS*.

Therefore, we investigated the status of MET expression together with PTEN expression and mutations of *BRAF* and *PIK3CA* in tumors of Japanese mCRC patients with wild-type *KRAS*. The main purpose of this study was to examine these genetic profiles for potential correlations with clinical response to anti-EGFR MoAb therapy.

## Materials and methods

### Patients

Clinical outcomes of anti-EGFR MoAb therapy were retrospectively analyzed for possible associations with the molecular features of tumors in mCRC patients. The study enrolled 91 patients who were treated at the Department of Gastroenterological Surgery and Medical Oncology, Kyorin University Hospital, between November 2008 and December 2012. All patients had presented with histologically confirmed mCRC and had been treated with salvage chemotherapy incorporating anti-EGFR MoAbs. Clinical features of the patients and pathological profiles of the tumors were obtained from patient medical records.

All patients received cetuximab- or panitumumab-based therapy for mCRC (11 as first-line, 29 as second-line, 39 as third-line, and 12 as fourth-line or greater). Cetuximab, as monotherapy or in combination with irinotecan, was administered intravenously (i.v.) at a loading dose of 400 mg/m^2^ over 2 h, followed by weekly doses administered at 250 mg/m^2^ over 1 h. Panitumumab was administered i.v. every 2 weeks at a dose of 6 mg/kg. Treatment was continued until disease progression (PD) or toxicity occurred. Clinical evaluation and tumor response was analyzed according to Response Evaluation Criteria in Solid Tumors (RECIST) [19]. This study was approved by the Research Ethics Committee, Hospital of Kyorin University School of Medicine.

### Mutational analysis of *KRAS*, *BRAF*, and *PIK3CA* by direct sequencing

Paraffin-embedded tissues (primary or metastatic) were sectioned at 10 μm thicknesses and mounted as three separate slides per tissue. The resulting slides were treated three times with xylene and then washed with ethanol. To minimize contamination by normal DNA, areas in which at least 70 % of the cells exhibited disease-specific pathology were dissected under a binocular microscope, from which DNA was extracted using the QIAamp FFPE Tissue Kit (QIAGEN). Segments of the *KRAS*, *BRAF*, and *PIK3CA* genes were amplified using gene-specific primers and subjected to direct DNA sequencing as previously described [4, 13, 20]. *KRAS* point mutations were screened for codons 12 and 13 within exon 2, two hot spots that cumulatively include >95 % of mutations in this gene [21]. *BRAF* mutations were screened for V600E within exon 15, in which >95 % of point mutations occur [7, 9]. *PIK3CA* mutations were screened within exons 9 and 20, in which >80 % of point mutations occur [4, 10, 12].

### Immunohistochemistry of PTEN and MET

PTEN and MET expression levels were evaluated by immunohistochemistry performed on 4-μm tissue sections of paraffin-embedded specimens. PTEN was assessed using the 17.A mouse MoAb (1:25 dilution; Neomarkers, Thermo Fisher Scientific Inc., Fremont, CA); MET was assessed using the SP44 rabbit MoAb (Spring Biosciences, Pleasanton, CA) [22, 23]. Negative controls were incubated with nonimmune solution instead of primary antibody. Endothelial cells and hepatocellular carcinoma cells were used as positive controls for PTEN and MET expression, respectively. The PTEN and MET staining intensities were evaluated by a pathologist (Y.O.) who was blinded to the diagnosis of individual patients.

To our knowledge, there currently are no validated scoring systems for interpretation of PTEN or MET staining intensity. Both PTEN and MET are localized primarily in the cytoplasm [11, 24, 25]; we therefore adopted a scoring system that has been used for other cytoplasmic proteins and is based on the intensity of immunoreactivity and percentage of stained cells [26, 27]. Specifically, intensity was scored according to a four-tier system: 0, no staining; 1, weak; 2, moderate; and 3, strong. An additional one, two, or three points were assigned if the percentage of positive cells was <25, 25–50 %, or >50 %, respectively [4, 11].

**Fig. 1:**
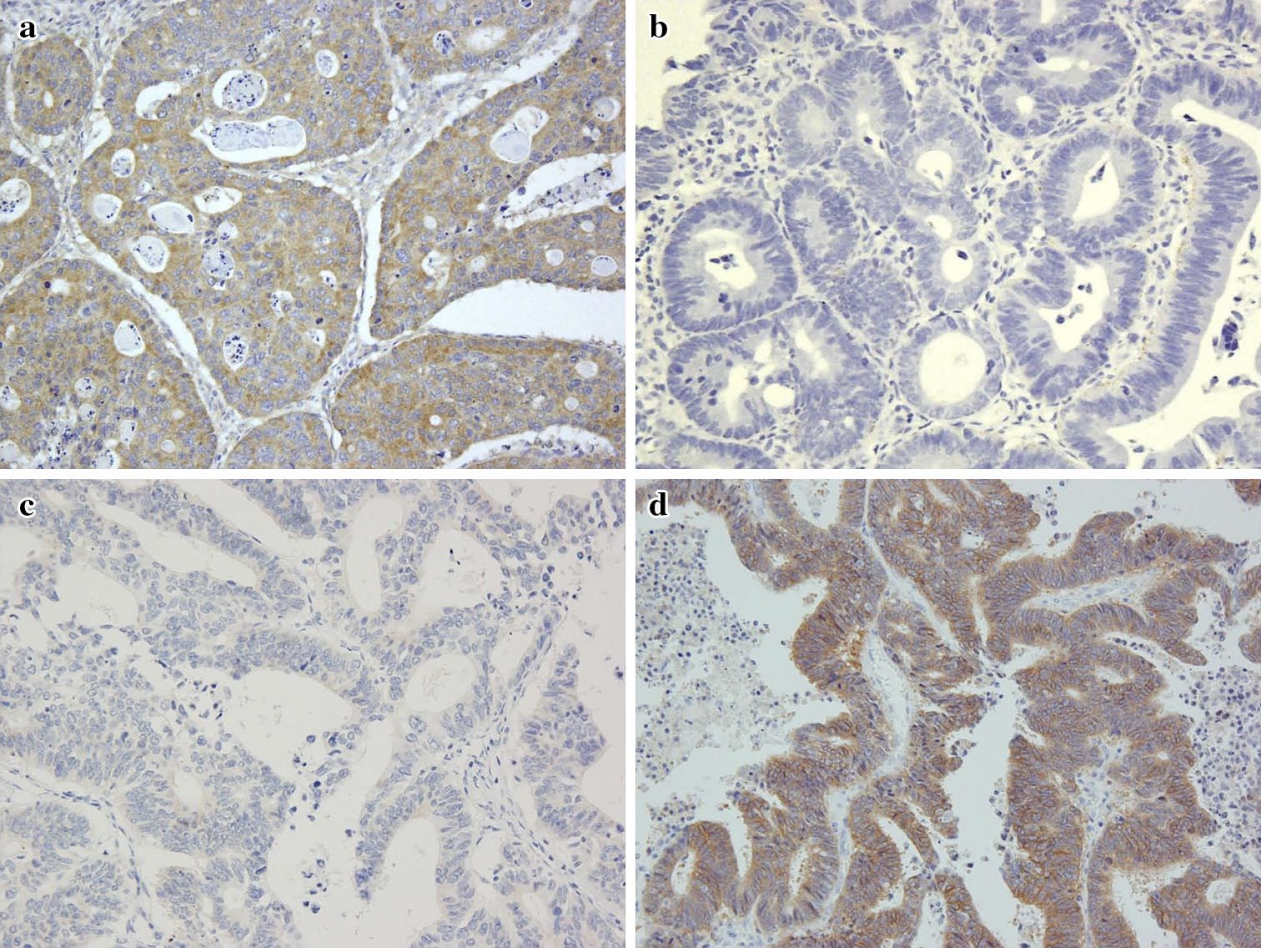
Representative examples of immunohistochemical staining in colorectal cancer. PTEN, normal expression (**a**) and loss of expression (**b**); MET, low expression (**c**) and overexpression (**d**)

We defined normal PTEN expression as a score of 4 or greater; scores of 0–3 were classified as loss of expression (Fig. 1a, b). We defined normal/low expression of MET as a score of 0–3; scores of 4 or greater were classified as MET overexpression (Fig. 1c, d).

### Statistical analysis

Comparison of categorical variables was performed with the *χ* ^2^ test or the Fisher’s exact test. The progression-free survival (PFS) and overall survival (OS) were calculated using the Kaplan–Meier method. Comparisons between different groups were performed using log-rank tests. To identify independent biomarkers, multivariate analyses were performed using a logistic regression model for response and a Cox regression model for PFS and OS. Two-tailed *P* values of <0.05 were considered significant. All analyses were performed using SPSS software (SPSS for Windows Version 15.0; SPSS Inc., Chicago, IL).

## Results

### Patient characteristics

All study patients were Japanese; they were 66 men and 25 women with a mean age of 67 years (range 38–85 years). At a median follow-up of 13.3 months (range 1.3–24.4 months), 78 patients (86 %) had progressed, and 41 patients (45 %) had died. Response to anti-EGFR therapy was evaluable in all patients. We observed no patients with complete response (CR), 27 with partial response (PR), 24 with stable disease (SD), and 40 with PD. Therefore, the overall RR was 29.7 %, and the disease control rate (DCR) was 56.0 %. In the whole group, PFS and OS were 3.9 and 13.3 months, respectively.

### *KRAS* mutational analysis

The mutational status of *KRAS* exon 2 was determined in all patients, with mutations detected in 24 patients (26.4 %). PR was observed in 26 patients with wild-type *KRAS* (38.8 %) and in one patient with mutation (4.2 %). RR and DCR were significantly lower in patients with *KRAS* mutations than in those with wild-type *KRAS*: For RR, the values were 4.2 versus 38.8 % (*P* < 0.001); for DCR, the values were 16.7 versus 70.2 % (*P* < 0.001). Median PFS was significantly shorter in patients whose tumors carried *KRAS* mutations than in those without such mutations (2.0 vs. 5.4 months; hazard ratio (HR) 1.67; 95 % confidence interval (CI) 1.29–2.14; *P* < 0.001; Supplementary Table 1, Supplementary Fig. 1A). Median OS was shorter in patients whose tumors carried *KRAS* mutations than in those without such mutations, although the difference was not statistically significant (9.9 vs. 13.4 months; HR 1.35; 95 % CI 0.96–1.85; *P* = 0.069; Supplementary Table 1, Supplementary Fig. 1B). Thus, our results indicated that *KRAS* mutations were reproducibly associated with less favorable outcomes for anti-EGFR MoAb therapy, consistent with the previous reports. Therefore, our further analyses focused primarily on patients with wild-type *KRAS* tumors. Table 1 summarizes the characteristics of the 67 patients who harbored tumors with wild-type *KRAS* genes.

**Table 1:** Characteristics of patients with wild-type *KRAS* (*n* = 67)

Characteristics	*n*	%
Age		
<70	41	61.2
>70	26	29.8
Gender		
Male	50	74.6
Female	17	25.4
Evaluated tumor		
Primary	63	94.3
Metastasis	4	5.7
Stage at diagnosis		
II and III	21	31.3
IV	46	68.7
Primary tumor location		
Cecum	1	1.5
Ascending colon	7	11.1
Transverse colon	3	4.5
Descending colon	2	3.0
Sigmoid colon	23	34.3
Rectum	32	47.8
Tumor differentiation		
Well/moderate	60	96.7
Poor	2	3.3
Site of metastasis		
Liver	49	73.1
Lung	33	49.3
Peritoneum	13	19.4
Others	13	19.4
EGFR-targeted therapies		
Cetuximab	25	37.3
Cetuximab + irinotecan	19	28.4
Cetuximab + FOLFIRI/FOLFOX	7	10.4
Panitumumab	13	19.4
Panitumumab + irinotecan	3	4.5
Anti-EGFR antibody administration line		
1st	10	14.9
2nd	23	34.3
3rd	28	41.8
4th or greater	6	9.0

*FOLFIRI* folinic acid, fluorouracil, and irinotecan, *FOLFOX* folinic acid, fluorouracil, and oxaliplatin

### *BRAF* mutational analysis

**Fig. 2:**
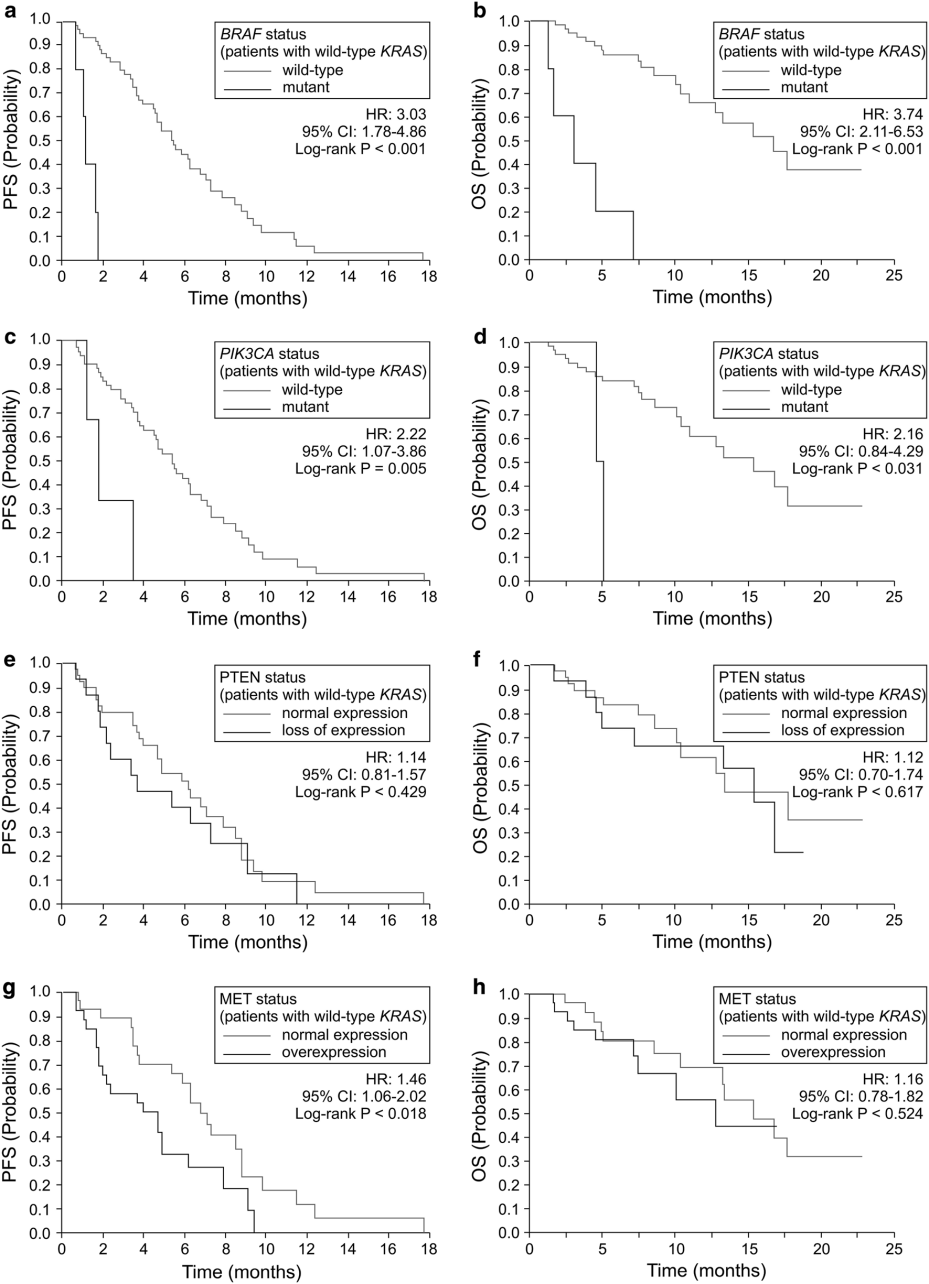
**a** Progression-free survival (*PFS*) and **b** overall survival (*OS*) in wild-type *KRAS* patients classified by *BRAF* mutational status. **c** Progression-free survival (*PFS*) and **d** overall survival (*OS*) in wild-type *KRAS* patients classified by *PIK3CA* mutational status. **e** Progression-free survival (*PFS*) and **f** overall survival (*OS*) in wild-type *KRAS* patients classified by PTEN expression status. **g** Progression-free survival (*PFS*) and **h** overall survival (*OS*) in wild-type *KRAS* patients classified by MET expression status

The mutational status of *BRAF* exon 15 was determined in all but one patient. Five (7.7 %) of 65 patients with wild-type *KRAS* harbored *BRAF* mutations, while none of 24 patients with *KRAS* mutation harbored mutations in *BRAF* (Supplementary Table 2). None of the *BRAF* mutant patients exhibited a response to MoAb therapy. In the patients with wild-type *KRAS*, the presence of the *BRAF* mutation was associated with a significantly reduced DCR (*P* = 0.002; Table 2). In this cohort, *BRAF* mutations were significantly associated with shorter PFS (1.2 vs. 5.5 months; HR 3.03; 95 % CI 1.78–4.86; *P* < 0.001; Table 3, Fig. 2a) and shorter OS (3.1 vs. 16.8 months; HR 3.74; 95 % CI 2.11–6.53; *P* < 0.001; Table 3, Fig. 2b). The PFS and OS of these patients were shorter than those of patients with *KRAS* mutations (median PFS 1.2 vs. 2.0 months; HR 1.70; 95 % CI 0.95–2.83; *P* = 0.037; and median OS 3.1 vs. 9.9 months; HR 0.52; 95 % CI 0.31–0.92; *P* = 0.009).

**Table 2:** Effect of biomarkers on RR and DCR of patients with wild-type *KRAS:* univariate analysis

	*n*	PR	SD	PD	RR (%)	*P*	DCR (%)	*P*
*BRAF*								
Wild	60	24	21	15	40		75.0	
Mutant	5	0	0	5	0	0.149	0	0.002
*PIK3CA*								
Wild	58	22	20	16	37.9		72.4	
Mutant	3	0	0	3	0	0.547	0	0.027
PTEN								
Normal expression	39	15	14	10	46.2		74.4	
Loss of expression	15	3	5	7	20.0	0.120	46.7	0.192
MET								
Normal/low expression	28	12	11	5	42.9		82.1	
Overexpression	26	9	5	12	34.6	0.586	53.9	0.040

*PR* partial response, *SD* stable disease, *PD* disease progression, *RR* response rate, *DCR* disease control rate

**Table 3:** Effect of biomarkers on PFS and OS in patients with wild-type *KRAS*: univariate analysis

	*n*	%	PFS			OS		
			Median (months)	HR (95 %CI)	*P*	Median (months)	HR (95 %CI)	*P*
*BRAF*								
Wild-type	60	92.3	5.5	3.03		16.8	3.74	
Mutant	5	7.7	1.2	(1.78–4.86)	<0.001	3.1	(2.11–6.53)	<0.0001
*PIK3CA*								
Wild-type	58	95.1	5.4	2.22		15.4	2.16	
Mutant	3	4.9	1.8	(1.07–3.86)	0.005	5.1	(0.84–4.29)	0.031
PTEN								
Normal expression	39	72.2	6.2	1.14		13.4	1.12	
Loss of expression	15	27.8	3.7	(0.81–1.57)	0.429	15.4	(0.07–1.74)	0.617
MET								
Normal/low expression	28	51.9	6.8	1.46		15.4	1.16	
Over expression	26	48.1	4.7	(1.06–2.02)	0.018	12.8	(0.73–1.82)	0.524

*PFS* progression-free survival, *OS* overall survival, *HR* hazard ratio, *CI* confidence interval

### *PIK3CA* mutational analysis

The mutational status of *PIK3CA* was determined in 84 patients. Mutations were detected in three (13 %) of 23 patients with *KRAS* mutations and three (5.2 %) of 58 patients with wild-type *KRAS* (*P* = 0.339; Supplementary Table 2). None of the *PIK3CA*-mutant patients exhibited a response to MoAb therapy. When analysis was limited to patients with wild-type *KRAS*, DCR was significantly associated with the *PIK3CA* mutational status (*P* = 0.027; Table 2). *PIK3CA* mutations also were significantly associated with shorter PFS (1.8 vs. 5.4 months; HR 2.22; 95 % CI 1.07–3.86; *P* = 0.005) and shorter OS (5.1 vs. 15.4 months; HR 2.16; 95 % CI 0.84–4.29; *P* = 0.031) (Table 3, Fig. 2c, d).

### PTEN immunohistochemical evaluation

Of 91 patients, 75 patients were evaluable for PTEN. Twenty-four patients (32 %) showed loss of PTEN expression in the cytoplasmic compartment of the tumor cells. No significant correlation was found between PTEN expression and *KRAS* mutational status (Supplementary Table 2). No significant association between PTEN expression and RR, DCR, PFS, or OS was detected in patients with wild-type *KRAS*, although patients with loss of PTEN tended to show lower RR and DCR than those with normal PTEN expression (Tables 2, 3, Fig. 2e, f).

### MET immunohistochemical evaluation

Of 91 patients, 75 patients were evaluable for MET, with overexpression of the protein detected in 36 samples (48 %) (Supplementary Table 1). As with PTEN, there was no correlation between MET expression and *KRAS* mutational status (Supplementary Table 2). In 54 wild-type *KRAS* patients evaluable for MET, MET overexpression was associated with lower DCR (53.9 % vs. 82.1 %, *P* = 0.040; Table 2). Furthermore, MET overexpression was associated with shorter PFS (4.7 vs. 6.8 months; HR 1.46; 95 % CI 1.06–2.02; *P* = 0.018; Table 3), but exhibited no correlation with OS (12.8 vs. 15.4 months; HR 1.16; 95 % CI 0.73–1.82; *P* = 0.524) in this patient subgroup (Table 3, Fig. 2g, h).

### Multivariate analyses

Multivariate analysis among patients with wild-type *KRAS* did not identify *BRAF* mutation, *PIK3CA* mutation, loss of PTEN expression, or MET overexpression as independent biomarkers for lower RR and DCR. However, the *BRAF* mutation and MET overexpression were identified as independent factors for shorter PFS among patients with wild-type *KRAS* (*BRAF*, *P* = 0.004; MET, *P* = 0.046) (Table 4). The *BRAF* mutation was also identified as an independent factor for shorter OS among patients with wild-type *KRAS* (OS, *P* = 0.001) (Table 5).

**Table 4:** Effect of biomarkers on PFS in patients with wild-type *KRAS*: multivariate analysis

Variables	Hazard ratio	P	95 % CI
*BRAF * (wild-type/mutant)	8.455	0.004	2.009–35.588
MET (normal/overexpression)	2.029	0.046	1.014–4.061

*CI* confidence interval

**Table 5:** Effect of biomarkers on OS in patients with wild-type *KRAS*: multivariate analysis

Variables	Hazard ratio	*P*	95 % CI
*BRAF* (wild-type/mutant)	9.648	0.001	2.473–37.648

*CI* confidence interval

## Discussion

In the present study, the MET expression, PTEN expression, and mutations of *BRAF* and *PIK3CA* in mCRC patients with wild-type *KRAS* were investigated in association with clinical response to anti-EGFR MoAb therapy. The most striking finding in this study was that MET overexpression was associated with lower DCR and shorter PFS in patients with wild-type *KRAS*. One previous study reported an association of MET overexpression with the response to anti-EGFR therapy in mCRC [13], although those researchers did not report the *KRAS* status of their study subjects. To the best of our knowledge, the present study is the first to clarify an association of MET overexpression with inferior clinical response to anti-EGFR MoAbs in mCRC patients with wild-type *KRAS*. The rate of MET overexpression in mCRC in the present study was 48 %, similar to those examined in the previous studies (17–79 %) [13, 28, 29].

MET is involved in many mechanisms of cancer proliferation and metastasis. MET contains a tyrosine kinase domain that initiates a range of signals to regulate various cellular functions [30]. MET can activate the RAS/RAF/MAPK and PTEN/PI3K/Akt pathways by itself or via EGFR transphosphorylation [15–18]. In fact, MET overexpression or genetic alteration has been shown to play a role in the pathogenesis of several tumor types. In CRC, overexpression of MET has been suggested to be associated with tumor progression [28, 31]. In addition, MET also contributes to cancer resistance against EGFR inhibitors through bypass signaling. In nonsmall cell lung cancer, amplification of MET is associated with resistance to gefitinib, the reversible EGFR tyrosine kinase inhibitor, via ErbB3 activation [17, 18, 32]. Resistance in that example is mediated by MET-ErbB3 transactivation, leading to restored signaling via the PI3K/AKT pathway [14]. Our present data revealed that MET overexpression is associated with shorter PFS, but not with altered OS, in mCRC patients with wild-type *KRAS* who received anti-EGFR MoAbs, suggesting that MET contributes to resistance against these therapies. If confirmed, these results attest to the feasibility of the recent development of MET-targeted agents against malignant diseases, a therapeutic approach that has already been reported in several phase I and II trials [33]. MET-targeted agents, alone or in combination with EGFR inhibitors, may offer the potential for improving patients’ outcome in mCRC.

This study also adds to the growing evidence that *BRAF* mutational status predicts efficacy of anti-EGFR therapy in mCRC patients with wild-type *KRAS.* Therefore, assessment of *BRAF* mutations before initiation of anti-EGFR therapy appears to be justified in this patient group. However, the clinical impact of *BRAF* gene testing depends on the prevalence of *BRAF* mutations. In this study, the frequency of *BRAF* mutations was 5 %, a value lower than that previously reported (7.9–16.6 %), possibly reflecting the fact that *BRAF* mutation is a negative prognostic marker that affects OS [34, 35]. In the present study, OS was shorter in *BRAF*-mutated patients compared with patients with wild-type *BRAF*, an observation that is consistent with the results of previous studies [4, 34, 35]. Therefore, some patients with *BRAF* mutations may not have survived long enough to be recruited into this study. The frequency of *BRAF* mutations might have been higher in a prospective study, which is expected to enroll all CRC patients. In addition, the prevalence of *BRAF* mutations was reported to be lower in Asian people than in Western people [34]. Taken together, these data suggest that the clinical relevance of analyzing *BRAF* mutation in Asian mCRC patients should be assessed by prospective studies in the future.

The frequency of *PIK3CA* mutations in the present study (8 %) was comparable to those in previous reports (7–18 %) [4, 10, 25]. Previous studies employing wild-type *KRAS* patients generally reported shorter median PFS or OS in *PIK3CA*-mutant patients than in patients with wild-type *PIK3CA* [4, 7, 10]. In concordance with these results, our patients with *PIK3CA* mutation showed significantly shorter PFS and OS and lower DCR than those without mutation. The present results confirmed that mutation of *PIK3CA* is also a predictive marker for response to anti-EGFR MoAb.

Low PTEN expression has been associated with shorter PFS in CRCs treated by anti-EGFR MoAbs in several reports [4, 11, 25], while no correlation was demonstrated in another report [8, 13]. We did not detect any association between PTEN expression status and clinical response to anti-EGFR MoAb therapy. This discrepancy may reflect differences in patient characteristics or study design, and notably, the distinct IHC scoring algorithms used in the present study. The use of a standardized methodology for assessment of PTEN expression would be crucial in the future studies.

This study has some limitations. Our study was performed retrospectively in a relatively small and heterogeneous population. The majority of our population (90 %) was treated with two or more chemotherapy regimens before anti-EGFR MoAb therapy. In addition, the anti-EGFR treatment protocols were heterogeneous. The discrepancy observed in the results between univariate and multivariate analyses might reflect these factors. Our findings therefore should be validated in subsequent prospective studies before they are applied in the clinical practice.

In conclusion, our data point out the usefulness of MET overexpression and mutations of *BRAF*, as a new predictive marker for response to anti-EGFR MoAbs in mCRC patients with wild-type *KRAS*. Using these two genes may be more useful for predicting the response to anti-EGFR MoAbs. These results support the emerging view that a comprehensive assessment of genetic alterations in EGFR signaling pathways will enable an accurate identification of patients who will benefit from anti-EGFR treatment and other molecular-targeting therapies, including MET inhibitors.

## Electronic supplementary material

Below is the link to the electronic supplementary material.

Supplementary material 1 (PDF 138 kb)

(A) PFS and (B) OS of 91 patients classified by *KRAS* mutational status (PDF 640 kb)
