# Supplementary material for: Overexpression of MET is a new predictive marker for anti-EGFR therapy in metastatic colorectal cancer with wild-type KRAS
Source: Cancer Chemother Pharmacol. 2014 Feb 6;73(4):749–57. doi: 10.1007/s00280-014-2401-4 (PMC3965831; doi:10.1007/s00280-014-2401-4)
Supplement: Supplementary file 1 — Supplementary material 1 (PDF 138 kb) [file 280_2014_2401_MOESM1_ESM.pdf]

Supplementary Table 1. Effect of biomarkers on PFS and OS: univariate analysis in all patients (n=91)

			PFS			OS		
	n	%	Median (months)	HR (95% CI)	*P*	Median (months)	HR (95% CI)	*P*
KRAS								
Wild-type	67	73.6	5.4			13.4		
Mutant	24	26.4	2.0	1.67 (1.29-2.14)	< 0.001	9.9	1.35 (0.96-1.85)	0.069
BRAF								
Wild-type	84	95.5	4.0			15.4		
Mutant	4	4.5	1.2	2.71 (1.54-4.34)	< 0.001	3.1	2.92 (1.67-4.63)	< 0.001
PIK3CA								
Wild-type	78	92.9	4.0			12.8		
Mutant	6	7.1	2.0	1.75 (1.08-2.60)	0.007	5.5	1.06 (0.51-1.79)	0.855
**PTEN**								
Normal expression	51	68.0	4.7			11.9		
Loss of expression	24	32.0	2.3	1.14 (0.88-1.47)	0.333	15.4	1.06 (0.75-1.48)	0.726
**MET**								
Normal/low expression	39	52.0	5.3			13.3		
Over expression	36	48.0	2.3	1.26 (0.98-1.62)	0.062	12.8	1.15 (0.82-1.60)	0.406

Abbreviations: PFS,progression free survival; OS, overall survival; HR, hazard ratio; CI, confidence interval

Supplementary Table 2. Relationships between *KRAS* mutation and other molecular biomarkers

	n	*KRAS* wild-type	*KRAS* mutant	*P*
BRAF				
Wild-type	84	60	24	
Mutant	5	5	0	0.318
PIK3CA				
Wild-type	78	58	20	
Mutant	6	3	3	0.339
**PTEN**				
Normal expression	51	39	12	
Loss of expression	24	15	9	0.209
**MET**				
Normal/low expression	39	28	11	
Over expression	36	26	10	0.967
