# Supplementary figures and images for: Overexpression of MET is a new predictive marker for anti-EGFR therapy in metastatic colorectal cancer with wild-type KRAS
Source: Cancer Chemother Pharmacol. 2014 Feb 6;73(4):749–57. doi: 10.1007/s00280-014-2401-4 (PMC3965831; doi:10.1007/s00280-014-2401-4)

Supplementary Figure 1

A

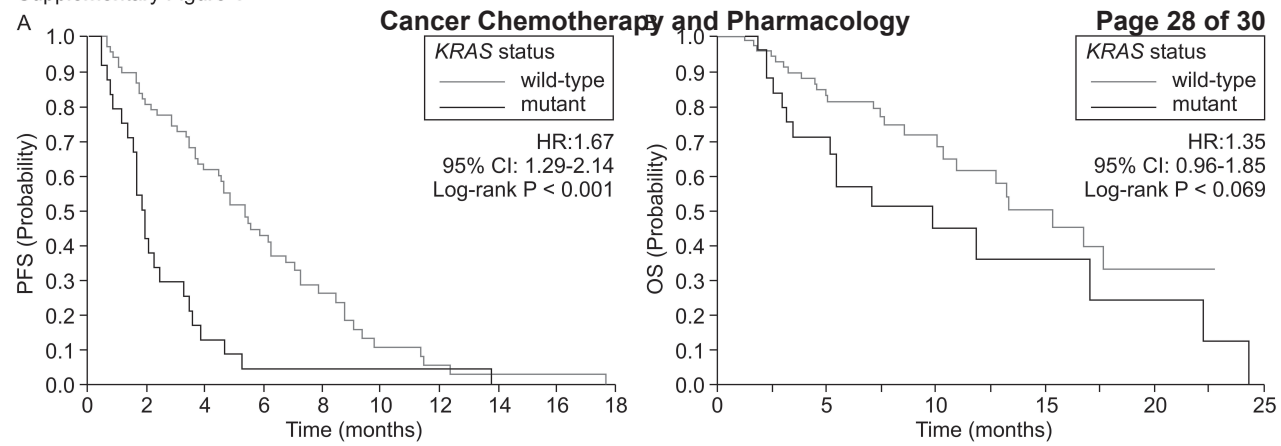

Supplement: Supplementary file 2 — (A) PFS and (B) OS of 91 patients classified by KRAS mutational status (PDF 640 kb) [file 280_2014_2401_MOESM2_ESM.pdf]
